# Licochalcone A Upregulates Nrf2 Antioxidant Pathway and Thereby Alleviates Acetaminophen-Induced Hepatotoxicity

**DOI:** 10.3389/fphar.2018.00147

**Published:** 2018-03-23

**Authors:** Hongming Lv, Qingfei Xiao, Junfeng Zhou, Haihua Feng, Guowen Liu, Xinxin Ci

**Affiliations:** ^1^Institute of Translational Medicine, The First Hospital of Jilin University, Changchun, China; ^2^Key Laboratory of Zoonosis, Ministry of Education, College of Veterinary Medicine, Jilin University, Changchun, China; ^3^Department of Nephrology, The First Hospital of Jilin University, Changchun, China

**Keywords:** licochalcone A, acetaminophen, hepatotoxicity, Nrf2, oxidative stress

## Abstract

Acetaminophen (APAP) overdose-induced fatal hepatotoxicity is majorly characterized by overwhelmingly increased oxidative stress while enhanced nuclear factor-erythroid 2-related factor 2 (Nrf2) is involved in prevention of hepatotoxicity. Although Licochalcone A (Lico A) upregulates Nrf2 signaling pathway against oxidative stress-triggered cell injury, whether it could protect from APAP-induced hepatotoxicity by directly inducing Nrf2 activation is still poorly elucidated. This study aims to explore the protective effect of Lico A against APAP-induced hepatotoxicity and its underlying molecular mechanisms. Our findings indicated that Lico A effectively decreased *tert*-butyl hydroperoxide (*t*-BHP)- and APAP-stimulated cell apoptosis, mitochondrial dysfunction and reactive oxygen species generation and increased various anti-oxidative enzymes expression, which is largely dependent on upregulating Nrf2 nuclear translocation, reducing the Keap1 protein expression, and strengthening the antioxidant response element promoter activity. Meanwhile, Lico A dramatically protected against APAP-induced acute liver failure by lessening the lethality; alleviating histopathological liver changes; decreasing the alanine transaminase and aspartate aminotransferase levels, malondialdehyde formation, myeloperoxidase level and superoxide dismutase depletion, and increasing the GSH-to-GSSG ratio. Furthermore, Lico A not only significantly modulated apoptosis-related protein by increasing Bcl-2 expression, and decreasing Bax and caspase-3 cleavage expression, but also efficiently alleviated mitochondrial dysfunction by reducing c-jun N-terminal kinase phosphorylation and translocation, inhibiting Bax mitochondrial translocation, apoptosis-inducing factor and cytochrome *c* release. However, Lico A-inhibited APAP-induced the lethality, histopathological changes, hepatic apoptosis, and mitochondrial dysfunction in WT mice were evidently abrogated in Nrf2^-/-^ mice. These investigations firstly implicated that Lico A has protective potential against APAP-induced hepatotoxicity which may be strongly associated with the Nrf2-mediated defense mechanisms.

## Introduction

The liver is vulnerable to multiple factors, including various drugs which result in liver injury or/and ALF (Bernal et al., 2015). Acetaminophen (*N*-acetyl-*p*-aminophenol) is a valid antipyretic and analgesic drug, whereas taking overdose APAP leads to severe liver injury (Jaeschke, 2015). As widely accepted mechanisms, the APAP-induced hepatotoxicity is associated with overproduction of reactive metabolite *N*-acetyl-*p*-benzoquinoneimine (NAPQI) predominantly via CYP2E1—the classical cytochrome P450 isozyme, which attributes to cause depletion of glutathione (GSH) and formation of ROS triggering oxidative stress to result in mitochondrial dysfunction, hepatocyte necrosis, and even liver injury (McGill and Jaeschke, 2013; Huo et al., 2017). Moreover, GSH and oxidized glutathione (GSSG) belong to the glutathione pool which is an essential redox buffer in various cells, and their ratios of change are thought to be an indicator of intracellular redox status (Schafer and Buettner, 2001), which plays an important role in the attenuation of APAP-induced ALF (Du et al., 2017). Accordingly, the inhibition of oxidative stress may play an essential role in attenuating APAP-induced ALF. Nuclear factor-erythroid 2-related factor 2 (Nrf2), a crucial transcription factor, is required to the improvement of progression of a variety of diseases, particularly those arising from oxidative stress (Kumar et al., 2014). Previous studies have suggested that Nrf2-deficiency mice have the greater severity than the wild-type (WT) mice in APAP-induced liver injury (Enomoto et al., 2001), targeting Nrf2 activation for effective prevention of hepatotoxicity.

Under basal conditions, Nrf2 is sequestered in the cytoplasm by Keap1 (Kelch-like ECH-associated protein 1, its repressor protein) (Malhotra et al., 2010). Upon exposure to stressors and inducers, however, Nrf2 dissociates from Keap1, enters to the nucleus and binds to the AREs, resulting in various antioxidant and detoxification genes expression, such as SOD, NAD (P) H: quinone oxidoreductase (NQO1), heme oxygenase-1 (HO-1) and the glutamate-cysteine ligase catalytic/modifier (GCLC/GCLM) subunit, which are essential for glutathione biosynthesis (Malhotra et al., 2010; Niso-Santano et al., 2010). Additionally, excessive ROS production and release stimulates a variety of oxidative stress responses, which further deteriorate mitochondrial dysfunction, cell damage and death to accelerate APAP-induced hepatotoxicity (Du et al., 2017; Wu et al., 2017). On the one hand, increasing evidence shows that cytochrome *c* is released into the cytoplasm, which is regarded as a marker of severe mitochondrial damage (Eleftheriadis et al., 2016). AIF is a caspase-independent death effector and mitochondrial intermembrane proteins similarly associated with mitochondrial dysfunction (Wang et al., 2012), whereas the release of AIF is induced initially by formation of a Bax (proapoptotic protein) pore (Bajt et al., 2008). Moreover, Bcl-2 family proteins, which is comprised of the pro-apoptotic protein Bax and the anti-apoptotic protein Bcl-2, are key regulators of mitochondrion-mediated apoptosis (Adams and Cory, 2007). On the other hand, cell apoptosis results in a change in a battery of protein expression levels, such as reducing caspase 3 activity, up-regulating antiapoptotic protein Bcl-2, and down-regulating the proapoptotic protein Bax (Yang et al., 2016). Furthermore, oxidative stress-induced MAP kinases activation, including resulting in JNK phosphorylation which translocates to mitochondria and further aggravates the mitochondrial oxidant stress (Hanawa et al., 2008; Saito et al., 2010) Intriguingly, abundant phytochemicals can enhance the Nrf2 signaling pathway activation to increase the capacity of antioxidation, which inhibit oxidative stress-induced cell apoptosis and mitochondrial dysfunction (Kulasekaran and Ganapasam, 2015; de Oliveira et al., 2017; Lv et al., 2017).

To date, abundant natural product-derived compounds, extensively distributed in vegetables, fruits and many medicinal plants, such as flavonoids, have been widely accepted as alternative and adjuvant medicines for various pathologies via induction of the Nrf2/ARE signaling pathway (Dixon and Steele, 1999; Gao et al., 2014; Kumar et al., 2014). Licochalcone A (Lico A), which is one of the primary flavonoids isolated from the root of the Xinjiang licorice Glycyrrhiza inflate, has various biological activities, including anti-inflammatory, antioxidant, antitumorigenic, and antimicrobial activities (Williams et al., 2004; Kwon et al., 2008). Furthermore, recent our previous studies discovered that Lico A could induce Nrf2-mediated defense mechanisms contributing to improve oxidative stress-induced cell injury in RAW 264.7 cells (Lv et al., 2015). Up to now, however, little is known about the protective effect of Lico A against APAP-induced hepatotoxicity *in vitro* and/or i*n vivo*. Consequently, we investigated the protective effect of Lico A on APAP-induced liver injury and the mechanisms, whether is involved in the regulation of Nrf2 signal pathway.

## Materials and Methods

### Reagents and Chemical

Licochalcone A (Lico A), purity > 98%, was provided by the Chengdu Herbpurify, Co., Ltd. (Chengdu, China). 3-(4, 5-dimethylthiazol-2-y1)-2, 5-diphenyltetrazolium bromide (MTT), Acetaminophen, *t*-BHP, and DMSO were purchased from Sigma–Aldrich (St. Louis, MO, United States). Fetal bovine serum (FBS), Dulbecco’s modified Eagle’s medium (DMEM), Penicillin and streptomycin were acquired from Invitrogen-Gibco (Grand Island, NY, United States). Antibodies against p-JNK, JNK, Keap1, Nrf2, HO-1, NQO1, GCLC, GCLM, cytochrome *c*, Bax, Bcl-2, caspase 3, Lamin B, β-actin were obtained from Cell Signaling (Boston, MA, United States) or Abcam (Cambridge, MA, United States). Additionally, ALT, AST, MDA, MPO, GSH, and SOD test kits were supplied by Nanjing Jiancheng Bioengineering Institute (Nanjing, China). The GSH and GSSG test kit was offered by Beyotime Biotechnology (Shanghai, China). All other chemicals were offered by Sigma–Aldrich (St. Louis, MO, United States), if not otherwise indicated.

### Animals

Wild-type and Nrf2^-/-^ (knockout) C57BL/6 mice were purchased from Liaoning Changsheng Technology Industrial, Co., Ltd. (Certificate SCXK2010-0001; Liaoning, China) and The Jackson Laboratory (Bar Harbor, ME, United States), respectively. All animals were raised under SPF-condition after feeding for several days. All animal experiments were performed according to the guide for the Care and Use of Laboratory Animals, which was published by the US National Institute of Health. This study was reviewed and approved by the Animal Welfare and Research Ethics Committee at Jilin University.

### Experimental Protocol

**FIGURE 1:**
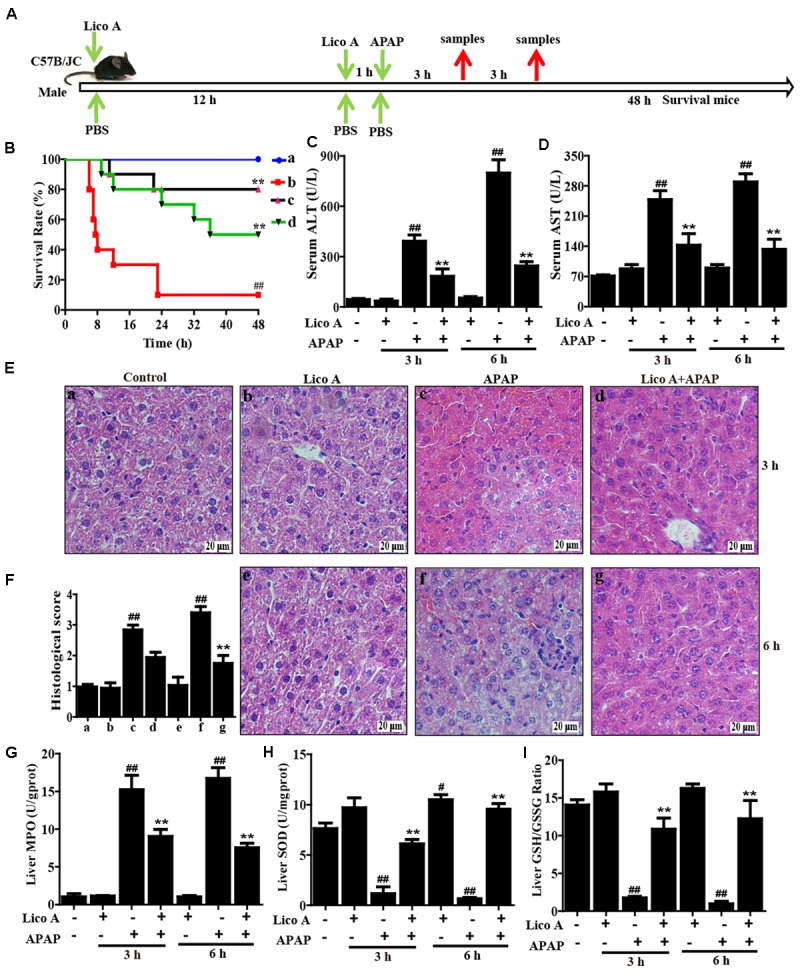
Protective effects of Lico A treatment on APAP-induced ALI. **(A)** Experimental protocol for APAP-induced ALI model, Lico A (50 or 100 mg/kg) was administered intraperitoneally to mice for twice at a 12-h (interval for 12 h), followed by subjected treatment with APAP (900 or 400 mg/kg). **(B)** The survival rates of the mice (*n* = 10/group) were observed within 48 h after APAP (900 mg/kg) exposure. (a) Control and Lico A group; (b) APAP group; (c) Lico A (100 mg/kg) + APAP; (d) Lico A (50 mg/kg) +APAP. **(C,D)** Sera were collected from the mice (*n* = 5/group) after exposure to APAP (400 mg/kg) for 3 and 6 h for measurement of the ALT and AST levels. **(E)** Representative histological sections of the livers (*n* = 5/group) were stained with hematoxylin and eosin (H & E)-stained (magnification × 400). **(F)** The stained sections were graded using a four-point scale from 0 to 3, with 0, 1, 2, and 3 representing no damage, mild damage, moderate damage, and severe damage, respectively. **(G–I)** Effects of Lico A on liver MPO, SOD, and GSH/GSSG levels. Similar results were obtained from three independent experiments. All data are presented as means ± SEM. ^#^*p* < 0.05 and ^##^*p* < 0.01 vs. Control group; ^∗∗^*p* < 0.01 vs. APAP group.

To induce APAP hepatotoxicity, two protocols were performed in our experiment as described **Figure 1A**. Protocol 1: WT mice were randomly divided into five groups: Control (PBS), Lico A only (100 mg/kg), APAP (900 mg/kg), Lico A (50 mg/kg) + APAP, and Lico A (100 mg/kg) + APAP groups were administered intraperitoneally. In brief, mice were intraperitoneally injected by Lico A (50 or 100 mg/kg) for twice (each time interval for 12 h), and at 1 h after the second dose of Lico A, followed by exposure to APAP (900 mg/kg). The survival rates of mice were observed for 48 h after APAP challenge. Protocol 2: WT mice were randomly separated into four groups: Control (PBS), Lico A only (100 mg/kg), APAP (400 mg/kg), and Lico A (100 mg/kg) + APAP groups were administered intraperitoneally. After APAP administration for 3 or 6 h, the animals were euthanized and then liver tissues and serum were harvested. In addition, to further investigate the protective effect of Lico A against APAP-induced hepatotoxicity, WT and Nrf2^-/-^ mice were respectively performed according to the above described protocol 2, but APAP (400 or 900 mg/kg) for 6 h. Subsequently, liver tissues samples and serum were collected and used for hematoxylin and eosin (H & E) staining, biochemical indexes assay and Western blotting assay.

### Histopathological Evaluation

Liver tissues were immersed in 10% neutral buffered formalin and fixed for 48 h, dehydrated in a series of graded ethanol, embedded in paraffin wax, and cut into 5-μm-thick sections. The paraffin-embedded sections were stained with hematoxylin and eosin (H & E) for pathological analysis under a light microscope. The histological changes were evaluated by a point-counting method for severity of hepatic injury using an ordinal scales in accordance with the methods as previous described (Camargo et al., 1997). The stained sections were graded as a four-point scale from 0 to 3 as follows: 0, 1, 2, and 3 represent no damage, mild damage, moderate damage and severe damage, respectively.

### Biochemical Indexes Assay

All mice were sacrificed at 3 or 6 h after APAP administration, liver and blood were collected for biochemical analysis. ALT and AST levels in serum and liver were measured using the corresponding detection kits in accordance with the manufacturer’s instructions. In addition, mice liver tissues were homogenized and dissolved in extraction buffer to analyze the MPO, GSH, MDA, and SOD levels according to the manufacturer’s instructions. All results were normalized by the total protein concentration in each sample.

### Cell Culture and MTT Analysis

A hepatoma-derived HepG2 cell line, obtained from the China Cell Line Bank (Beijing, China), were cultured in DMEM medium supplemented with 10% FBS, 100 U/Ml of penicillin, 100 U/mL of streptomycin and 3 mM glutamine at 37°C in a humidified atmosphere containing 5% CO_2_. In all experiments, cells were allowed to acclimate for 24 h before any treatments. Moreover, WT and Nrf2^-/-^ HepG2 cells (1 × 10^4^ cells/well) were treated with Lico A (3.7 μM) for 1 h, and exposed to APAP (15 mM) for 24 h. Cell viability was measured by MTT assay in accordance with the manufacturer’s instructions. MTT (5 mg/mL) was added to the cells and incubated for another 4 h; the supernatant was removed, and DMSO was added to each well to lyse the cells. Then, the absorbance was measured at 570 nm.

### Intracellular ROS Measurement

To detect intracellular ROS production, HepG2 cells were seeded into 96-well plates (1 × 10^4^ cells/well) for 24 h, and recovered in serum-free DMEM for 6 h. Then, the cells were subjected to different dosages of Lico A (1.85 or 3.7 μM) for 18 h, and incubated with 50 μM of DCFH-DA for 30 min prior to stimulation with *t*-BHP. DCF fluorescence intensities were assessed by flow cytometry or a multi-detection reader at excitation and emission wavelengths of 485 and 535 nm, respectively.

### Apoptosis Quantification

HepG2 cells (2.5 × 10^5^ cells/well) were maintained in 12-well plates for 24 h and were then subjected to different dosages of Lico A (1.85 or 3.7 μM) for 18 h followed by exposing to *t*-BHP (5 mM) for an additional 1 h. Cells were washed twice with ice-cold PBS, collected and centrifuged at 1500 rpm for 5 min at 4°C. Next, cells were measured by Hoechst 33342 and propidium iodide staining, and the percentages of apoptosis and necrosis were assessed using flow cytometry.

### JC-1 Assay for Mitochondrial Membrane Potential (MMP)

Mitochondrial membrane potential (MMP) (ΔΨ) was measured by JC-1 staining. Briefly, HepG cells were seeded into 12-well plates at a density of 2 × 10^5^ cells/well for 24 h. Subsequently, the cells were subjected to different dosages of Lico A (1.85 or 3.7 μM) for 18 h, followed by exposure to *t*-BHP (5 mM) for an additional 1 h. Next, the cells were washed with PBS and incubated with JC-1 (10 μg/mL) at 37°C in the dark for 20 min. The ΔΨm was analyzed for intracellular fluorescence by a multi-detection reader.

### CRISPR/Cas9 Knockout of Nrf2 Gene (Qi et al., 2017)

HepG2 cells were cultured in 12-well plates at the density of 3 × 10^5^ cells/well for 24 h. The plasmids of expressing Cas9 with Nrf2-sgRNA and puromycin resistant gene were co-transfected into HepG2 cells using Viafect transfection reagent (Promega). At 48 h after transfection, cells were added puromycin at a concentration of 2 μg/mL and harvested for immunoblotting analysis with Nrf2 antibody. After 7 days, cells were cultured in a 96-well plates (1 cell/well).

### Total RNA Extraction and qPCR

Total RNA from cells was isolated using Trizol reagent according to the procedure described by the manufacturer. After the concentration of RNA was determined by spectrophotometer, 1 μg of RNA was transformed into cDNA using Prime-Script RT-PCR kit (Takara). PCR reactions were carried out using the SYBR green working solution and quantitatively measured with the Applied Biosystems 7300 real-time PCR system and software (Applied Biosystems, Carlsbad, CA, United States). The following thermal cycler parameters were used: 95°C for 10 min, followed by 40 cycles of 95°C for 10 s, and 60°C for 30 s. Gene expression changes were calculated by the comparative Ct method and the values were analyzed by normalizing with β-actin mRNA expression.

### ARE Promoter Activity

HepG2 cells were seeded in 96-well plates (1 × 10^4^ cells/well) until approaching approximately 75% confluence. Then, pGL4.37 and pGL4.74 plasmids were transfected into cells using Viafect transfection reagent in accordance with the manufacturer’s protocol (Invitrogen, Carlsbad, CA, United States). After Lico A (3.7 μM) treatment for different time periods or Lico A (0.925, 1.85, or 3.7 μM) treatment for 6 h, a dual-luciferase reporter assay system (Dual-Glo^®^ Luciferase Assay System) was used to detect and analyze ARE-driven promoter activity.

### Preparation of Nuclear and Cytosolic Fractions

The nuclear and cytoplasmic extracts were prepared using an NE-PER Nuclear and Cytoplasmic Extraction Reagents kit (Pierce Biotechnology, Rockford, IL, United States) in accordance with the manufacturer’s instructions. All steps were carried out on ice or at 4°C unless stated otherwise.

### Isolation of Subcellular Fractions

Fresh liver tissues were homogenized in ice cold isolation buffer [pH 7.4, containing 1 mM ethylene glycol tetraacetic acid, 22 mM mannitol, 70 mM sucrose, 10 mM EDTA, 2.5 mM 4-(2-hydroxyethyl)-1-piperazineethanesulfonic acid (HEPES), and 0.1% bovine serum albumin]. The homogenate was centrifuged at 2500 *g* for 10 min. The supernatant was then centrifuged at 20,000 *g* for 10 min, the pellet was mitochondria, and the supernatant was preserved as the cytosolic fraction. The mitochondrial and cytosolic fractions were used for detection.

### Western Blotting Analysis

Liver tissues were collected 3 or 6 h after APAP challenge. Total protein was extracted from the liver tissues using a protein extract kit according to the manufacturer’s protocol. Protein concentrations were tested by the BCA method. Equal amounts of proteins (20 μg) were separated by a 10% SDS-polyacrylamide gel and transferred onto a polyvinylidene difluoride (PVDF) membrane. The membrane was blocked with 5% (w/v) non-fat milk for 2 h. Then, the membrane was incubated with primary antibody and secondary antibody. Finally, the membranes were visualized by the ECL Western blotting detection system in accordance with the manufacturer’s instruction and band intensities were quantified using Image J gel analysis software. All experiments were performed in triplicate.

### Statistical Analysis

All data referenced above were expressed as the means ± SEM and analyzed using SPSS19.0 (IBM). Comparisons between experimental groups were conducted using one-way ANOVA, whereas multiple comparisons were made using the LSD method. Statistical significance was defined as *p* < 0.05 or *p* < 0.01.

## Results

### Lico A Treatment Attenuated APAP-Induced Acute Liver Failure (ALF) Mice

To investigate whether Lico A could protect against APAP-induced ALF in mice, the survival rate firstly was observed within 48 h after APAP exposure. As shown in **Figure 1B**, the mice died at 7 h after APAP injection, and survival rate reached 5% at 48 h, whereas pretreatment with Lico A (100 mg/kg) effectively increased survival rate up to 80%. Given levels of ALT and AST related to the liver injury, the ALT and AST levels in serum of APAP-induced mice were measured. Our results indicated that ALT and AST levels in serum were significantly increased by APAP administration when compared with Control group. However, this increase was markedly reduced Lico A pretreatment, indicating that Lico A treatment efficiently protected against APAP-induced ALF (**Figures 1C,D**). Meanwhile, histological analysis of mice liver sections in the APAP group showed remarkably disturbed architecture, such as hepatocyte necrosis, hemorrhage, and neutrophil infiltration. However, APAP-induced liver alterations were efficiently relieved by Lico A treatment, which was measured by the liver injury score (**Figures 1E,F**). Next, our further results found that APAP not only evidently increased MDA formation and MPO level, but also obviously decreased SOD and GSH content in liver of mice, whereas Lico A pretreatment dramatically inhibited these effects-induced by APAP (**Figures 1G–I**).

### Lico A Treatment Alleviated APAP-Induced Mitochondrial Dysfunction and Apoptosis in Mice

**FIGURE 2:**
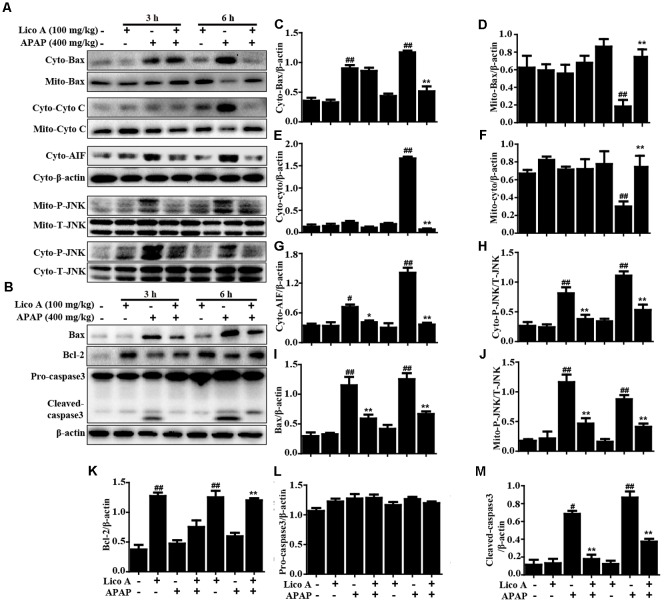
Effect of Lico A treatment on APAP-induced mitochondrial dysfunction and apoptosis in mice. Liver tissues were collected from the mice 3 h or 6 h after APAP challenge and analyzed by Western blotting analysis. **(A)** Immunoblot analysis showed the levels of Bax, AIF, cytochrome *c* (Cyto C) and P-JNK in the cytoplasmic and mitochondria isolated from liver tissues harvested at 3 or 6 h post-APAP (400 mg/kg) injection. **(B)** Liver tissues lysates were examined for caspase-3 cleavage using Western blotting analysis. **(C–M)** Quantification of relative protein expression was performed by densitometric analysis. Similar results were obtained from three independent experiments. All data are presented as means ± SEM (*n* = 5/group). ^#^*p* < 0.05 and ^##^*p* < 0.01 vs. Control group; ^∗^*p* < 0.05 and ^∗∗^*p* < 0.01 vs. APAP group.

Due to mitochondrial dysfunction and cell apoptosis were regarded as two crucial factors in APAP-induced liver injury, our studies examined whether Lico A pretreatment could inhibit APAP-induced mitochondrial dysfunction and cell apoptosis. Indeed, APAP (400 mg/kg) significantly induced Bax mitochondrial translocation, the release of AIF and cytochrome c, and JNK activation, whereas these effects are effectively inhibited by Lico A pretreatment (100 mg/kg). In addition, our results uncovered that APAP challenge not only obviously increased apoptotic protein caspase-3 cleavage and Bax expression, but also decreased antiapoptotic protein Bcl-2 expression. However, these phenomena were remarkably blocked by Lico A administration, suggesting that Lico A pretreatment could attenuate APAP-induced mitochondrial dysfunction and cell apoptosis (**Figure 2**).

### Lico A Treatment Upregulated Antioxidant Signaling Pathways in APAP-Induced ALF

**FIGURE 3:**
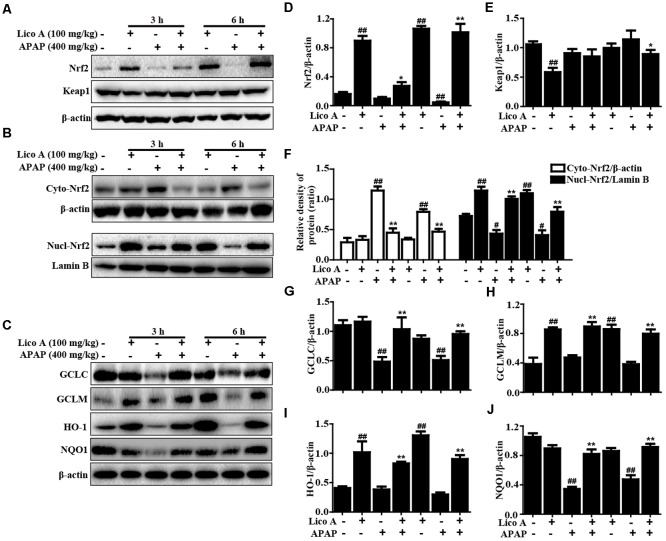
Effects of Lico A treatment on antioxidant signaling pathways in APAP-induced ALI. Mice at 3 h or 6 h after APAP challenge, liver tissues were collected and analyzed by Western blotting. **(A–C)** Effects of Lico A on Keap1, Nrf2, GCLC, GCLM, HO-1, and NQO1 protein expression and Nrf2 nuclear translocation. **(D–J)** Quantification of relative protein expression was performed by densitometric analysis. β-Actin and Lamin B were acted as an internal control. Similar results were obtained from three independent experiments. All data are presented as means ± SEM (*n* = 5/group). ^#^*p* < 0.05 and ^##^*p* < 0.01 vs. Control group; ^∗^*p* < 0.05 and ^∗∗^*p* < 0.01 vs. APAP group.

Oxidative damage is also one of major factor in APAP-induced ALF mice. Hence, Nrf2-mediated signaling pathway, as an important antioxidant pathway, was firstly taken into account whether Lico A pretreatment could upregulate Nrf2 antioxidative signaling pathway in APAP-induced ALF. In this study, APAP challenge mildly suppressed Nrf2, HO-1, GCLM protein expression, but strongly inhibited GCLC and NQO1 protein expression, whereas these changes were completely reversed by Lico A pretreatment when compared with the control group. Furthermore, we further found that Lico A pretreatment could result in a decrease in the cytoplasmic levels and a concomitant increase in the nuclear levels of Nrf2 in APAP-induced ALF (**Figure 3**).

### Lico A Treatment Inhibited *t*-BHP- and APAP-Induced Hepatotoxicity, Oxidative Stress, and Cell Apoptosis in HepG2 Cells

**FIGURE 4:**
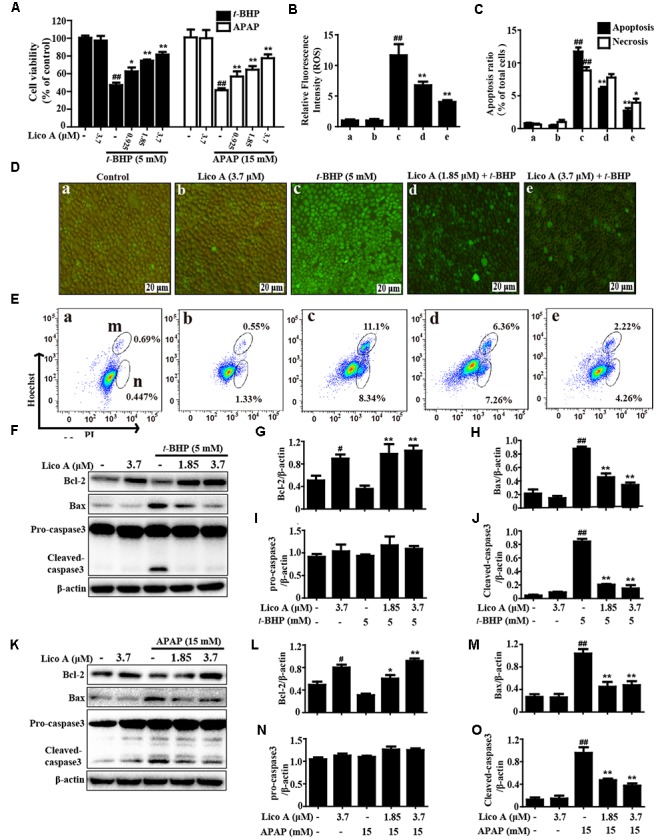
Effect of Lico A treatment on *t*-BHP- and APAP-induced hepatotoxicity, oxidative stress, and apoptosis in HepG2 cells. **(A)** HepG2 cells were subjected to Lico A (0.925, 1.85, or 3.7 μg/mL) for 18 h, subsequently stimulated with *t*-BHP (5 mM) for 3 h or APAP (15 mM) for 24 h, cell viability was determined by MTT assay. In addition, cells were treated with or without Lico A for 18 h, and were stained with 50 μM of DCFH-DA for 30 min, and subsequently exposed to *t*-BHP (5 mM) for 5 min to induce the ROS generation. **(B,D)** DCF fluorescence intensities were detected by a fluorescent microscope. **(C,E)** The percentage of cell apoptosis and necrosis were measured using flow cytometry. (m and n) Represent apoptosis and necrosis, respectively. Moreover, HepG2 cells were subjected to Lico A (1.85 or 3.7 μM) for 18 h, subsequently stimulated with *t*-BHP (5 mM) for 1 h or APAP (15 mM) for 3 h. **(F,K)** The expression of apoptosis-related proteins (Bax, Bcl-2, and caspase-3) were determined by Western blotting analysis. **(G–J,L–O)** Quantification of relative protein expressions were performed by densitometric analysis and β-actin was acted as an internal control. Similar results were obtained from three independent experiments. All results were expressed as means ± SEM of three independent experiments. ^#^*p* < 0.01 and ^##^*p* < 0.01 vs. the Control group; ^∗^*p* < 0.05 and ^∗∗^*p* < 0.01 vs. the *t*-BHP or APAP group.

To explore the potential inhibitory of effect of Lico A on hepatotoxicity, oxidative stress and cell apoptosis, we employed *t*-BHP- and APAP-induced toxicity in HepG2 cells, and cell viability was measured by an MTT assay. Our results showed that Lico A (0.925, 1.85, and 3.7 μM) protected the cells from *t*-BHP- and APAP-triggered oxidative cytotoxicity (**Figure 4A**). Simultaneously, Lico A treatment apparently suppressed *t*-BHP-triggered the generation of ROS, indicating that Lico A exposure could efficiently inhibit *t*-BHP-induced oxidative damage in HepG2 cells (**Figures 4B,D**). Moreover, *t*-BHP also significantly resulted in cell death by promoting apoptosis and necrosis in total cells, whereas Lico A (1.85 and 3.7 μM) significantly lessened *t*-BHP-induced cell apoptosis and necrosis (**Figures 4C,E**). Thus, to further certify the possible mechanisms of the role of Lico A in anti-apoptosis, we detected its effect on *t*-BHP- and APAP-induced apoptosis-related proteins. Our investigations showed that Lico A dramatically increased Bcl-2 protein expression and decreased the form of cleaved-caspase-3 and Bax protein expression in *t*-BHP- and APAP-induced HepG2 (**Figures 4F–O**).

### Lico A Treatment Improved *t*-BHP- and APAP-Induced Mitochondrial Dysfunction in HepG2 Cells

**FIGURE 5:**
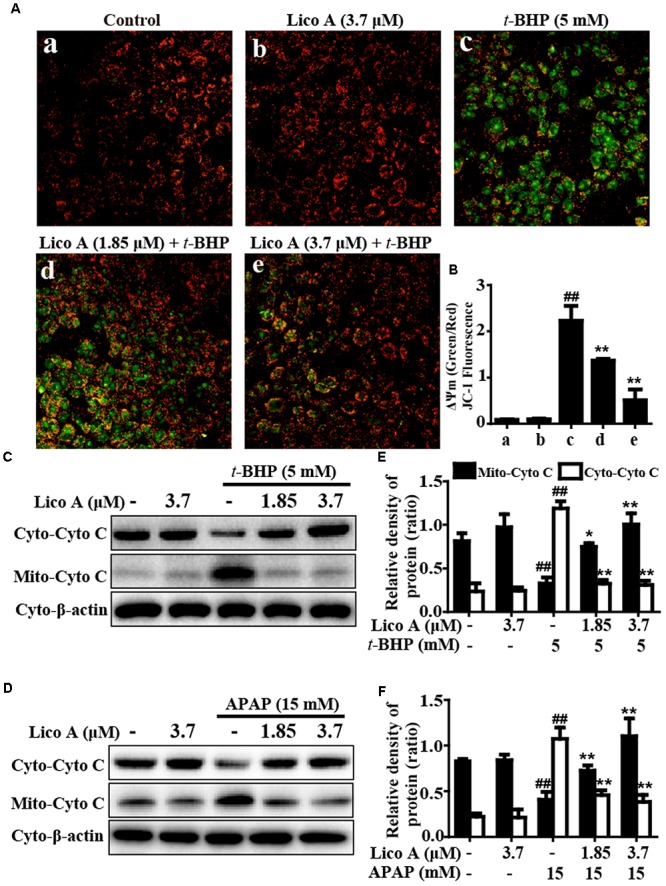
Effects of Lico A on *t*-BHP- and APAP-induced mitochondrial dysfunction in HepG2 cells. Cells were treated with various concentrations of Lico A (1.85 or 3.7 μM) for 18 h, and subsequently exposed to *t*-BHP (5 mM) for 1 h. **(A,B)** The effects of Lico A on mitochondrial membrane potential (MMP) were tested using the JC-1 method and were determined by a fluorescent microscope. Moreover, HepG2 cells were subjected to Lico A (1.85 or 3.7 μM) for 18 h, subsequently stimulated with *t*-BHP (5 mM) for 1 h or APAP (15 mM) for 3 h. **(C,D)** Cytochrome *c* (Cyto C) was measured in cytosolic and mitochondrial cells using Western blotting analysis. **(E,F)** Quantification of cytochrome *c* protein expression was performed by densitometric analysis and β-actin was acted as an internal control. Similar results were obtained from three independent experiments. All results were expressed as means ± SEM of three independent experiments. ^##^*p* < 0.01 vs. the Control group; ^∗^*p* < 0.05 and ^∗∗^*p* < 0.01 vs. the *t*-BHP or APAP group.

Moreover, to further elucidate whether Lico A suppressed *t*-BHP- and APAP-induced apoptosis associated with the mitochondria pathway. In the present study, Fluorescent microscope analysis showed that Lico A (1.85 and 3.7 μM) effectively reduced *t*-BHP-induced the replacement of red fluorescence by green JC-1 monomers, which represents the loss of MMP (ΔΨm) and mitochondrial dysfunction (**Figures 5A,B**). Given that mitochondria cytochrome *c* transfers to cytoplasm displays mitochondrial dysfunction, the effect of Lico A on *t*-BHP- and APAP-induced cytochrome *c* expression was examined by Western blotting analysis. Our results indicated that various concentrations of Lico A (1.85 and 3.7 μM) decreased *t*-BHP- and APAP-induced the release of cytochrome *c* into cytoplasm in HepG2 cells (**Figures 5C–F**).

### Lico A Treatment Enhanced Keap1-Nrf2/ARE Antioxidative Signaling Pathway in HepG2 Cells

**FIGURE 6:**
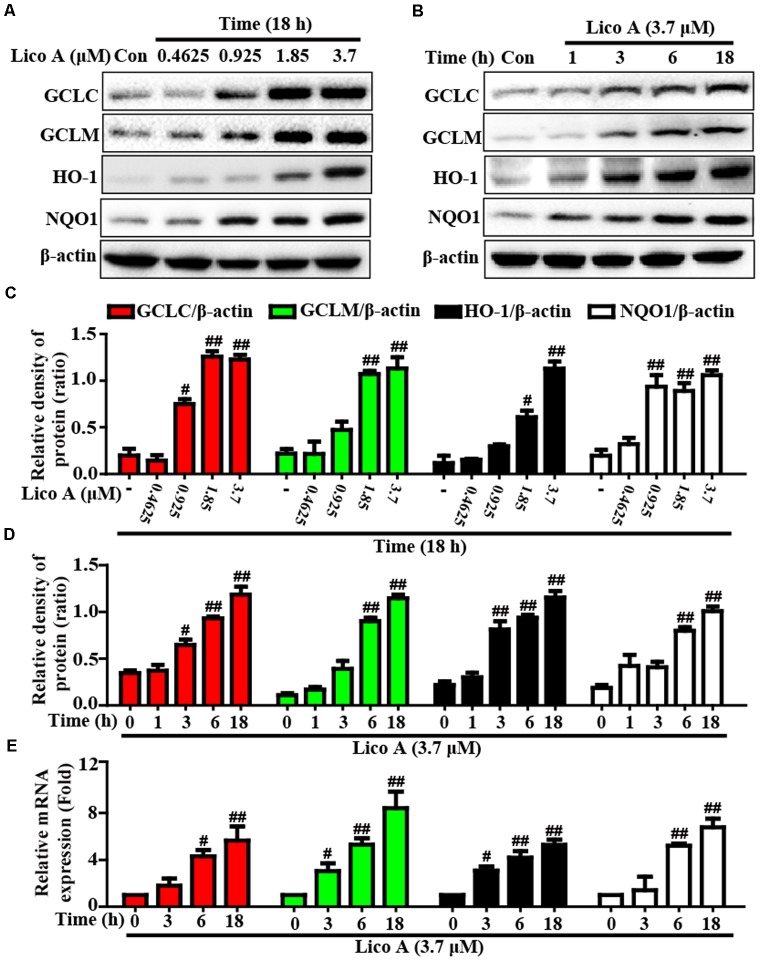
Effects of Lico A exposure on the upregulation of Nrf2-mediated antioxidant signaling pathway in HepG2 cells. **(A)** HepG2 cells were treated with different concentrations of Lico A (0.4625, 0.925, 1.85, or 3.7 μM) for 18 h, and **(B)** cells were exposed to Lico A (3.7 μM) for four time points (1, 3, 6, or 18 h). Protein expressions of GCLC, GCLM, HO-1, and NQO1 were measure by Western blotting analysis. **(C,D)** Quantification of GCLC, GCLM, HO-1, and NQO1 protein expressions were performed by densitometric analysis and β-actin was acted as an internal control. **(E)** Cells were exposed to Lico A (3.7 μM) for three time points (3, 6, or 18 h). Effects of Lico A on GCLC, GCLM, HO-1, and NQO1 genes expression. Total RNA was extracted from HepG2 cells and genes expression was quantified using real-time PCR. Similar results were obtained from three independent experiments. All results were expressed as means ± SEM of three independent experiments. ^#^*p* < 0.05 and ^##^*p* < 0.01 vs. the Control group.

**FIGURE 7:**
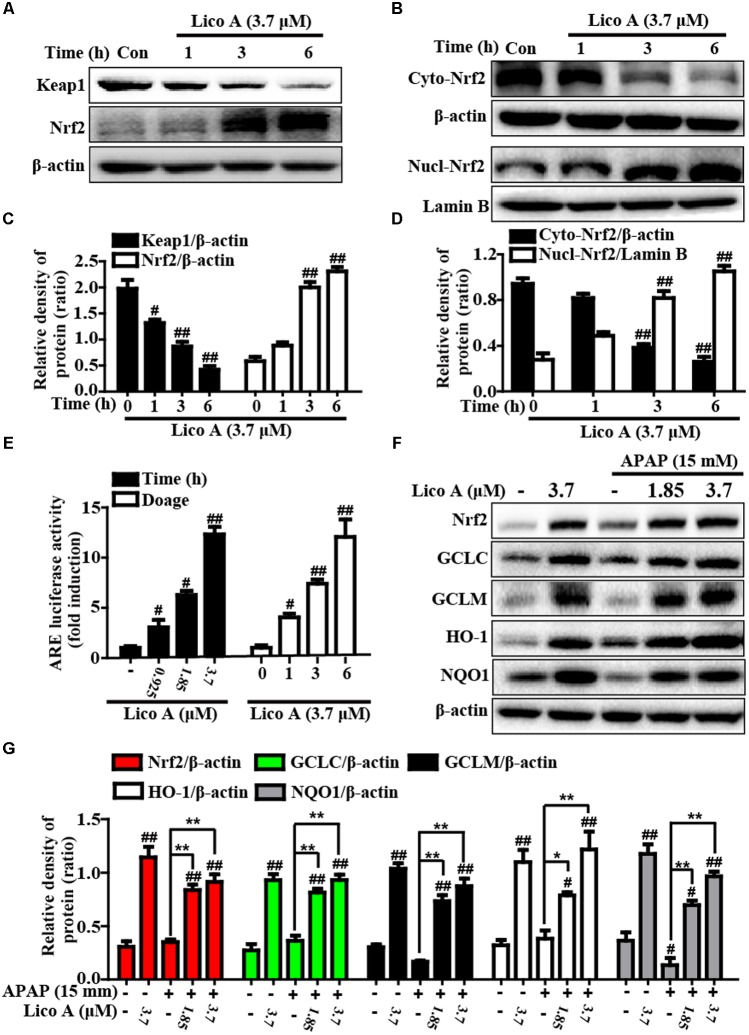
Effects of Lico A exposure on Keap1-Nrf2/ARE signaling pathway in HepG2 cells. **(A)** Cells were exposed to various concentration of Lico A (3.7 μM) for four time points (1, 3, or 6 h), and the total protein were determined by Western blotting analysis. **(B)** Cells were exposed to Lico A (3.7 μM) for three time points (1, 3, or 6 h), and the nuclear and cytoplasmic levels of Nrf2 were examined by Western blotting analysis. **(E)** The luciferase plasmids pGL-ARE and pRL-TK was transiently transfected into cells for 24 h and subsequently exposed to 3.7 μM Lico A for three time points (1, 3, or 6 h) or exposed to Lico A (0.925, 1.85, or 3.7 μM) for 6 h. ARE luciferase activity was detected by a dual-luciferase reporter assay system. Additionally, HepG2 cells were subjected to Lico A (1.85 or 3.7 μM) for 1 h, subsequently stimulated with and without APAP (15 mM) for 6 h. **(F)** Effects of Lico A on Nrf2, GCLC, GCLM, NQO1, and HO-1 protein expression. **(C,D,G)** The relative density of protein was performed by densitometric analysis; β-actin and Lamin B were acted as an internal control, respectively. Similar results were obtained from three independent experiments. All results were expressed as means ± SEM of three independent experiments. ^#^*p* < 0.05 and ^##^*p* < 0.01 vs. Control group; ^∗^*p* < 0.05 and ^∗∗^*p* < 0.01 vs. APAP group.

More importantly, Keap1-Nrf2/ARE signaling pathway is essential for inhibition of oxidative stress and plays an important role in amelioration of APAP-induced hepatotoxicity. Based on the above outcomes and our previous studies (Lv et al., 2015), we further attempted to probe into whether Lico A treatment could up-regulate Keap1-Nrf2/ARE signaling pathway in HepG2. In our studies, Lico A pretreatment evidently enhanced GCLC, GCLM, HO-1, and NQO1 genes expression in different dosages and periods (**Figure 6**). Meanwhile, our further results unveiled that Lico A (3.7 μM) efficiently the induced the Keap1 degradation and enhanced total Nrf2 protein expression, but beyond that it could result in an increase in the nuclear levels and a concomitant decrease in the cytoplasmic levels of Nrf2 in a time-dependent manner (**Figures 7A–D**). Moreover, because the increased Nrf2 expression in the nucleus is necessary for ARE activation, the ARE-luciferase plasmid were transiently transfected into the cells, and ARE activation was detect by changes in luciferase activity. This result discovered that Lico A also dramatically strengthened ARE-driven luciferase activity in a time- and dose-dependent manner (**Figure 7E**). Furthermore, HepG2 cells are pretreated with or without Lico A, and exposed to APAP, and then Nrf2, GCLC, GCLM, HO-1, and NQO1 protein expression were measure by Western blotting analysis. These results found that APAP minimally induced Nrf2, GCLC, GCLM, HO-1, and NQO1 protein expression, whereas Lico A pretreatment could markedly enhanced these proteins expression (**Figures 7F,G**).

### Lico A Treatment-Ameliorated APAP-Induced Hepatotoxicity Is Dependent Upon Nrf2 in HepG2 Cells and Mice

**FIGURE 8:**
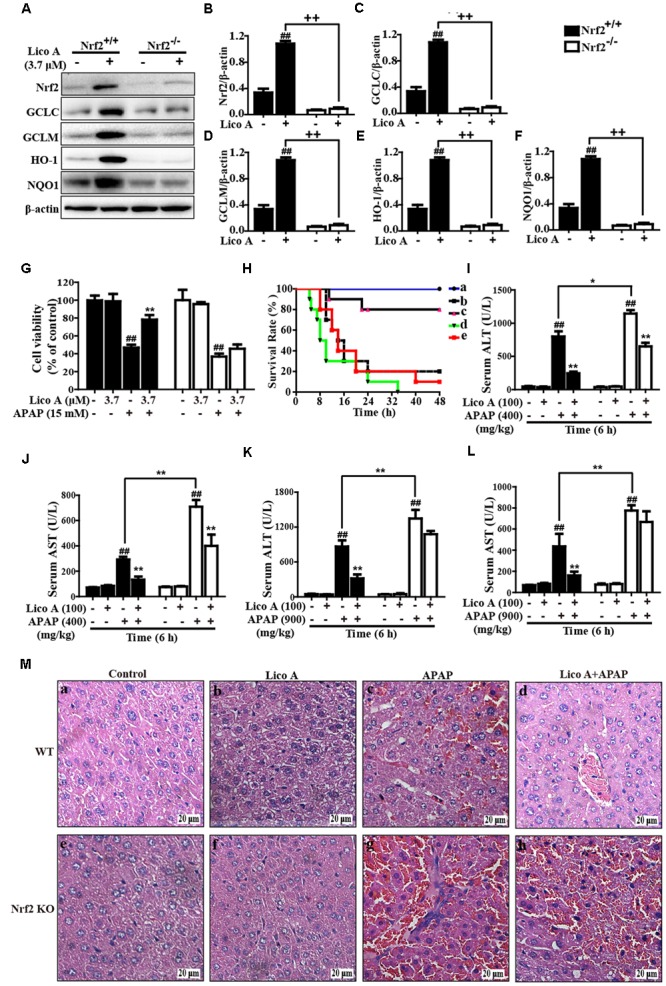
Protective effects of Lico A-meditated Nrf2 on APAP-induced hepatotoxicity *in vitro* and *in vivo*. **(A)** WT and Nrf2^-/-^ HepG2 cells were subjected to Lico A (3.7 μM) for 6 h and these antioxidant protein expressions were measured by Western blotting analysis. **(B–F)** The relative density of protein was performed by densitometric analysis; β-actin was acted as an internal control. **(G)** WT and Nrf2^-/-^ HepG2 cells were pretreatment with and without Lico A (3.7 μM) for 6 h, and then exposed to APAP (15 mM) for 24 h, cell viability was determined by MTT assay. Moreover, WT and Nrf2^-/-^ mice were intraperitoneally injected Lico A (100 mg/kg) with mice for twice at a 12-h (interval for 12 h), followed by subjected treatment with APAP (900 mg/kg). **(H)** The survival rates of the mice (*n* = 10/group) were observed within 48 h after APAP exposure. (a) Control and Lico A group; (b) APAP group; (c) Lico A (100 mg/kg) + APAP. **(I,J)** Sera were collected from the mice after exposure to APAP (400 mg/kg) for 6 h for measurement of the ALT and AST levels. **(K,L)** Sera were collected from the mice after exposure to APAP (400 or 900 mg/kg) for 6 h for measurement of the ALT and AST levels. **(M)** Livers (*n* = 5) from each experimental group were processed for histological evaluation at 6 h after the APAP (900 mg/kg) challenge. Similar results were obtained from three independent experiments. All data are presented as means ± SEM. ^##^*p* < 0.01 vs. Control group; ^∗^*p* < 0.05 and ^∗∗^*p* < 0.01 vs. APAP group; ^++^*p* < 0.01 vs. Lico A + APAP group.

Based on the outcomes described above, we hypothesized that the protective effect of Lico A-exhibited against APAP-induced hepatotoxicity is dependent on Nrf2 upregulation. Next, to verify the hypothesis, Nrf2 was knocked out in HepG2 cells using the CRISPR/Cas9 gene editing system, and WT/Nrf2^-/-^ mice were conducted in the experiment. In the present study, our work measured GCLC, GCLM, HO-1, and NQO1 protein expressions in WT and Nrf2^-/-^ HepG2 cells. Our results revealed that Lico A-mediated expression of GCLC, GCLM, HO-1, and NQO1 were almost abolished in Nrf2^-/-^ HepG2 cells (**Figures 8A–F**). Our further experiments determined the effect of Lico A (3.7 μM) on APAP-stimulated cell viability in WT and Nrf2^-/-^ HepG2 cells. This result showed that the inhibitory effect of Lico A on APAP-stimulated cytotoxicity were mostly blocked in Nrf2^-/-^ HepG2 cells (**Figure 8G**). Furthermore, the effect of Lico A (100 mg/kg) on the lethality of APAP-induced ALF was observed in WT and Nrf2^-/-^ mice, finding that Nrf2^-/-^ mice appeared to be more vulnerable to APAP-induced lethality than WT mice, which declines from about 80% to roughly 20% (**Figure 8H**). Subsequently, our results uncovered that Lico A-inhibited plasma levels of ALT and AST induced by APAP administration in WT mice were significantly impeded in Nrf2^-/-^ mice at 900 mg/kg rather than 400 mg/kg of APAP for 6 h (**Figures 8I–L**). Consequently, we observed histopathological changes at 900 mg/kg of APAP administration in WT and Nrf2^-/-^ mice and found that Lico A treatment alleviated severe histopathological changes in WT mice were apparently abrogated in Nrf2^-/-^ mice (**Figure 8M**). These investigations demonstrated that Lico A-displayed hepatoprotective effect may be involved in targeting Nrf2 pathway activation.

### Lico A Treatment Suppressed APAP-Induced Mitochondrial Dysfunction and Cell Apoptosis by Upregulation of Nrf2 in Mice

**FIGURE 9:**
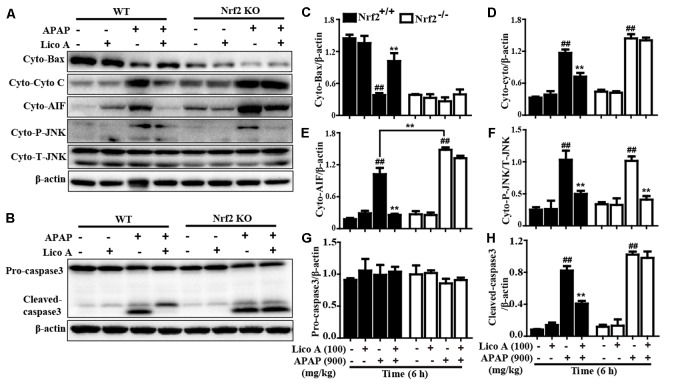
Effects of Lico A-meditated Nrf2 on APAP-induced mitochondrial dysfunction and apoptosis in mice. WT and Nrf2^-/-^ mice were intraperitoneally injected Lico A (100 mg/kg) with mice for twice at a 12-h (interval for 12 h), followed by subjected treatment with APAP (900 mg/kg) for 6 h. **(A)** Effects of Lico A on expressions of Cyto-Bax, Cyto-cytochrome *c*, Cyto-AIF, and Cyto-P-JNK protein. Additionally, **(B)** effects of Lico A on expressions of caspase-3 protein. **(C–H)** The relative density of protein was performed by densitometric analysis; β-actin was acted as an internal control. Similar results were obtained from three independent experiments. All data are presented as means ± SEM (*n* = 5/group). ^##^*p* < 0.01 vs. Control group; ^∗∗^*p* < 0.01 vs. APAP group.

Last but not least, our further studies confirmed whether Lico A-alleviated APAP-induced mitochondrial dysfunction and apoptosis is directly associated with upregulation of Nrf2. As presented in **Figure 9**, Lico A treatment remarkably inhibited APAP-induced Bax mitochondrial translocation, the release of AIF and cytochrome *c* in WT mice, which were obviously abrogated in Nrf2^-/-^ mice. However, Lico A (100 mg/kg) treatment still effectively suppressed APAP-activated JNK phosphorylation in Nrf2^-/-^ mice. Meanwhile, Lico A significantly decreased APAP-induced caspase-3 cleavage protein expression, whereas this phenomenon was obviously impeded in Nrf2^-/-^ mice. Taken together, our experimental results indicated that Lico A plays a significant role in the improvement of APAP-induced hepatotoxicity via mitochondrial dysfunction and cell apoptosis, which may be dependent upon induction of Nrf2.

## Discussion

Acute overdoses of acetaminophen-induced fatal hepatotoxicity results in 1000s of emergency department visits and hospitalizations, which is characterized by exceedingly increased oxidative stress (Nourjah et al., 2006; McGill et al., 2012). Although *N*-acetylcysteine (NAC) has been approved clinical antidote against APAP overdose since in the 1970s, recent reports showed that it is only effective during the early APAP overdose patients rather than in the later period of the injury peak (Polson et al., 2005; Larson, 2007). Accordingly, it is still necessary for APAP overdose-induced patients to discover some effective undeveloped drugs and investigate their potential molecular mechanism. Importantly, growing evidence reveals that Nrf2, a key coordinator of oxidative stress, plays an indispensable role in the amelioration of acute liver injury (Jiang et al., 2016; Palliyaguru et al., 2016). Previous numerous studies indicated that Licochalcone A (Lico A) not only exhibits various biological activities, such as antioxidant, antitumorigenic, anti-inflammatory, and antimicrobial properties (Haraguchi et al., 1998; Kwon et al., 2008), and but also induces Nrf2 activation to further alleviate *t*-BHP-triggered oxidative damage (Lv et al., 2015). In the present study, we focused on investigating whether Lico A could upregulate Nrf2 signaling pathway which contributes to prevent from APAP-induced ALF mice.

Under normal conditions, ALT and AST mostly exist in liver cells; however, these enzymes are transferred through the cell membrane into the serum when liver cells are impaired (Brodsky et al., 2009), indicating severe injury of liver functions. In the present study, Lico A treatment significantly reduced lethality, the ALT and AST levels in the APAP-induced ALF. On the one hand, it is generally accepted that induced lipid peroxidation and lessened functioning of non-enzymatic or enzymatic antioxidant defense systems are the primary characteristics of APAP-induced hepatotoxicity (Jadeja et al., 2015). On the other hand, administration of APAP to mice induces malondialdehyde and myeloperoxidase formation and decreases in hepatic levels of SOD and glutathione, which aggravates oxidative damage to further lead to liver tissue damage (Wu et al., 2017). To date, the consensus of opinion is that the metabolite of APAP results in depletion of GSH and formation of GSSG that contributes to cause mitochondrial oxidant stress and ultimately promote liver injury (Li et al., 2017). In addition, previous abundant researches indicated that APAP-induced hepatic structural integrity by displaying serious destruction of hepatic architecture, necrosis, and infiltration of inflammatory cells (Hasanein and Sharifi, 2017). In this study, Lico A treatment effectively suppressed the MDA and MPO formation, decreased the GSH and SOD depletion, increased the GSH/GSSG ratio, and attenuated pathological changes, indicating that Lico A treatment dramatically alleviated APAP-induced oxidative injury in mice with ALF. Accumulating evidence has showed that APAP-intoxicated liver tissues not only induces hepatic apoptosis by decreasing the Bcl-2/Bax ratio and increasing caspase-3 activities, but also provokes mitochondrial dysfunction by enhancing release of cytochrome *c* and AIF from the mitochondria, Bax translocation to mitochondria (Bajt et al., 2008; Kumari and Kakkar, 2012; Dong et al., 2014). Our findings discovered that Lico A treatment efficiently inhibited Bax mitochondrial translocation, the release of AIF and cytochrome *c* and caspase-3 cleavage. Furthermore, previous many reports have indicated that inhibiting or knockouting hepatocyte-specific JNK in mice could alleviate mitochondrial oxidative stress and reduce hepatic apoptosis and liver injury (Hanawa et al., 2008; Saito et al., 2010). Lico A treatment obviously inhibited APAP-induced JNK phosphorylation and mitochondrial translocation. These investigations implied that Lico A hepatoprotective effect is related to reduce hepatic apoptosis and deactivate mitochondrial dysfunction *in vivo* and *in vitro*. Next, our further studies explored the mechanism of the protective effect of Lico A against APAP-induced oxidative stress, hepatic apoptosis and mitochondrial dysfunction in ALF mice.

Nrf2, as a multiple signaling pathways coordinator, improves oxidative stress-induced cell apoptosis and mitochondrial dysfunction (Lv et al., 2017) and attenuates a variety of acute and chronic diseases by regulating various antioxidant genes GCLC, GCLM, NQO1, and HO-1 expressions (Song et al., 2015; Jiang et al., 2016). In this work, Lico A significantly enhanced GCLC, GCLM, NQO1, and HO-1 genes expression and induced Nrf2 nuclear translocation in the mice with APAP-induced ALF. Meanwhile, our findings indicated that various dosages and periods of Lico A exposure dramatically increased antioxidant genes and the expression of HO-1, GCLM, GCLC and NQO1 in HepG2 cells, whereas these effects were blocked in Nrf2^-/-^ HepG2 cells. Moreover, previous reports suggested that activated-Nrf2 is released from Keap1 and translocated into the nucleus, whereupon it sequentially binds to ARE in the promoter region of its target genes (Nguyen et al., 2003). In the present study, Lico A effectively-induced a decrease of Keap1 and an increase of Nrf2 in total cell lysates, which was associated with promoting the nuclear translocation of Nrf2 and strengthening ARE luciferase activity in a time- and dose-dependent manner in HepG2. These observations revealed that Lico A treatment could efficiently-induced Nrf2 antioxidative signaling pathway activation in HepG2 cells and mice with APAP-induced ALF. Given all of these results, to further elucidate whether Lico A-inhibited APAP-excited oxidative damage, hepatic apoptosis and mitochondrial dysfunction is directly involved in Nrf2 activation, Nrf2 deficient mice and Nrf2^-/-^ HepG2 cells were used as a tool for exploring an underlying connection. Our findings noticed that Lico A-reduced APAP-induced cytotoxicity and mice death were effectively abrogated in Nrf2^-/-^ HepG2 cell and mice. Surprisingly, Lico A still evidently decreased an increase of ALT and AST levels induced by APAP at 400 mg/kg in Nrf2^-/-^ mice, whereas Lico A did not exhibit this effect at 900 mg/kg of APAP in Nrf2^-/-^ mice. Accordingly, we speculated the possible reason for a low dose of APAP-induced hepatotoxicity could be alleviated by Lico A via activating and/or inhibiting another signaling pathway, but Lico A-attenuated a high dose APAP-induced hepatotoxicity is dependent upon Nrf2 activation. Next, 900 mg/kg of APAP was used to induce hepatotoxicity in the following experiments. Our further observations discovered that Lico A-improved severe histopathological changes in WT mice were obviously blocked in Nrf2^-/-^ mice. Furthermore, Lico A-mediated a decreases of Cyto-Bax and an increase of Cyto-AIF and Cyto-Cyto C protein expressions in WT mice were efficiently inhibited in Nrf2^-/-^ mice. In addition to these, Lico A-suppressed an increase of caspase3 cleavage in WT mice were dramatically impeded in Nrf2^-/-^ mice. However, Lico A-inhibited Cyto-P-JNK levels in WT mice were not blunted in Nrf2^-/-^ mice, indicating that Lico A-inhibited JNK activation did not rely on Nrf2 upregulation. Collectively, our experimental results provided a support that Lico A is essential for amelioration of APAP-induced hepatic injury by inhibition of oxidative stress, hepatic apoptosis, and mitochondrial dysfunction which may be involvement in upregulation of Nrf2 pathway.

## Conclusion

**FIGURE 10:**
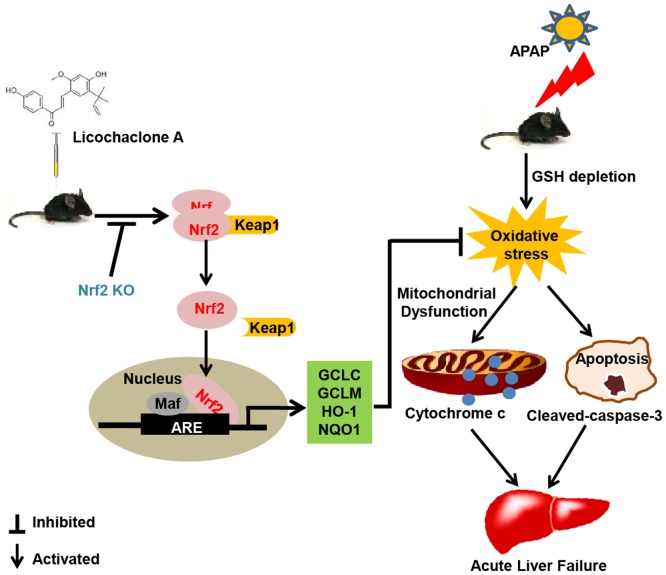
Lico A-mediated Nrf2 signaling pathway protects APAP-induced ALF via inhibition of mitochondrial dysfunction and apoptosis. Licochalcone A (Lico A) can induce the Nrf2 activation, which contribute to up-regulation of Nrf2 signaling pathway resulting in expression of abundant antioxidant genes. Furthermore, Lico A effectively reduced APAP-induced mitochondrial dysfunction and apoptosis by the inhibition of Bax mitochondrial translocation, AIF and cytochrome *c* release, and cleaved-caspase-3 formation, which were obviously abrogated in Nrf2^-/-^ mice.

As shown in **Figure 10**, the investigations of this study indicated a protective effect of Lico A against acetaminophen-induced hepatotoxicity by inhibiting oxidative damage, mitochondrial dysfunction, and apoptosis. The underlying mechanisms may be nearly involved in induction of GCLC, GCLM, HO-1 and NQO1 expression, increase of GSH/GSSG ratio, reduction of Bax mitochondrial translocation, inhibition of AIF and cytochrome *c* release, and suppression of apoptosis-related protein caspase-3 cleavage expression, which may be strongly dependent upon Lico A-mediated Nrf2 defense mechanisms. Therefore, the study provides beneficial evidence for the application of Lico A in protecting the liver from oxidative stress-induced mitochondrial dysfunction and apoptosis during APAP-induced ALF.

## Author Contributions

HL wrote the paper and performed the experiments. QX performed the experiments. JZ, HF, and GL analyzed the data. XC and GL contributed to design the experiments. All authors contributed to final approval of the article.

## Conflict of Interest Statement

The authors declare that the research was conducted in the absence of any commercial or financial relationships that could be construed as a potential conflict of interest. The reviewer XT and handling Editor declared their shared affiliation.

## Abbreviations

AIF: apoptosis-inducing factor
ALF: acute liver failure
ALT: alanine transaminase
APAP: acetaminophen
ARE: antioxidant response element
AST: aspartate aminotransferase
*t*-BHP: *tert*-butyl hydroperoxide
JNK: c-jun N-terminal kinase
Lico A: licochalcone A
MDA: malondialdehyde
MPO: myeloperoxidase
Nrf2: nuclear factor-erythroid 2-related factor 2
ROS: reactive oxygen species
SOD: superoxide dismutase.
